# Complexity of type-specific 56 kDa antigen CD4 T-cell epitopes of *Orientia tsutsugamushi* strains causing scrub typhus in India

**DOI:** 10.1371/journal.pone.0196240

**Published:** 2018-04-26

**Authors:** Arunachalam Ramaiah, Munegowda C. Koralur, Gregory A. Dasch

**Affiliations:** Rickettsial Zoonoses Branch, Centers for Disease Control and Prevention, Atlanta, GA, United States; University of Minnesota, UNITED STATES

## Abstract

*Orientia tsutsugamushi* (Ots) is an obligate, intracellular, mite-transmitted human pathogen which causes scrub typhus. Understanding the diversity of Ots antigens is essential for designing specific diagnostic assays and efficient vaccines. The protective immunodominant type-specific 56 kDa antigen (TSA) of Ots varies locally and across its geographic distribution. TSA contains four hypervariable domains. We bioinformatically analyzed 345 partial sequences of TSA available from India, most of which contain only the three variable domains (VDI-III) and three spacer conserved domains (SVDI, SVDII/III, SVDIII). The total number (152) of antigenic types (amino acid variants) varied from 14–36 in the six domains of TSA that we studied. Notably, 55% (787/1435) of the predicted CD4 T-cell epitopes (TCEs) from all the six domains had high binding affinities (HBA) to at least one of the prevalent Indian human leukocyte antigen (HLA) alleles. A surprisingly high proportion (61%) of such TCEs were from spacer domains; indeed 100% of the CD4 TCEs in the SVDI were HBA. TSA sequences from India had more antigenic types (AT) than TSA from Korea. Overall, >90% of predicted CD4 TCEs from spacer domains were predicted to have HBA against one or more prevalent HLA types from Indian, Korean, Asia-Pacific region or global population data sets, while only <50% of CD4 TCEs in variable domains exhibited such HBA. The phylogenetically and immunologically important amino acids in the conserved spacer domains were identified. Our results suggest that the conserved spacer domains are predicted to be functionally more important than previously appreciated in immune responses to Ots infections. Changes occurring at the TCE level of TSA may contribute to the wide range of pathogenicity of Ots in humans and mouse models. CD4 T-cell functional experiments are needed to assess the immunological significance of these HBA spacer domains and their role in clearance of Ots from Indian patients.

## Introduction

The Alphaproteobacterium *Orientia tsutsugamushi* (Ots) is an obligate, intracellular, slow-growing, mite-transmitted human pathogen which causes scrub typhus [1]. Scrub typhus is distributed across the Asia-Pacific region and the classic hotspot area stretches from Pakistan in the west to Australia in the east and north to Korea and Japan, coincident with the ranges of its primary vectors [2–11]. Many antigenic variants are recognized for Ots [2–3, 9, 12–14]. This Ots heterogeneity exists locally and across its geographic distribution [2]. The antigenic types of Ots related to the Gilliam, Karp, and Kato strains were historically recognized by serological techniques, and these strains were often used as prototype strains [2]. Five additional antigenic types (TA678, TA686, TA716, TA763, TH1817) were first defined in Thailand with cross-protection assays and used for direct fluorescent typing of additional isolates [14]. Later Shimokoshi, Kawasaki and Kuroki were distinguished by serological cross-tests with strain-specific polyclonal or monoclonal antibodies and genetic typing in Japan [13, 15, 16]. Boryong strain from South Korea has been an important new type because of its predominant clinical importance and use as the reference strain for studies on scrub typhus in Korea [17]. Although antigenic variation occurs in different Ots surface antigens, the best studied is the immunodominant 56 kDa major protein located on the Ots outer membrane, thus called the type-specific antigen (TSA) of this organism [1–2, 12–22]. This antigen is the primary target for genetic and monoclonal typing of Ots and can elicit immunity as a subunit vaccine in both protein and DNA constructs for Ots. The open reading frame of TSA encodes ~1600-bp for 516–541 amino acids [1–2]. The protein is generally involved in host cell invasion through the binding of fibronectin [23] and has four variable domains (VD) I–IV based on the TSA amino acid variability characterized from a large number of isolates [2].

Typically, antigenic variation arising from host immune pressure can lead to immune escape; to what extent this contributes to Ots persistence in an infected host is poorly understood but it does not appear to be due to in vivo *de novo* generation of antigenic diversity as occurs in bacterial relatives like *Anaplasma marginale*. Antigenic heterogeneity has been an important issue that has hampered development of effective diagnostic assays and scrub typhus vaccines based on the TSA [1, 2]. The major histocompatibility complex (MHC) molecules display the peptide/epitope derived from Ots on the human cell surface for recognition by the appropriate T-cells. MHC is a major driving force in shaping Ots immune escape, resulting in T-cell epitope (TCE) mutations. However, if this immune selection occurs with TSA, it most likely occurs in infected rodent hosts because human hosts are a dead end for *Orientia* infections. Hosts like rats and bandicoots with immunologically selected Ots variants must pass these new isolates to the chigger vector but acquisition by uninfected chiggers occurs at a very low, and nearly undetectable rate under laboratory conditions. Recombination has been suggested to occur in TSA evolution [17, 24], but this requires two genetically different strains and a susceptible chigger vector where the new genotype can be sustained transovarially. Low frequencies of mixed TSA Ots types have been detected in infected chiggers [25]. Nonetheless, regardless of how the genetic diversity of Ots TSA arose, a better understanding of the contemporary quality and quantity of antigenic diversity of TSA in Ots is a prerequisite for designing specific serological diagnostic assays, and for developing more efficient vaccines. We therefore investigated the prevalence of TSA antigenic variants in Ots from India [19, 20] and predicted the immunodominant CD4 TCEs that would be associated with significant HLA-DR binding affinity. In this context, we recognized the importance of analyzing the different TSA domains to find those immunogenic features and interactions which appear to be most conserved in most Indian individuals experiencing scrub typhus infections.

## Materials and methods

### Analysis of sequences of TSA from *O*. *tsutsugamushi* strains and prediction of CD4 T-cell epitopes

**Fig 1:**
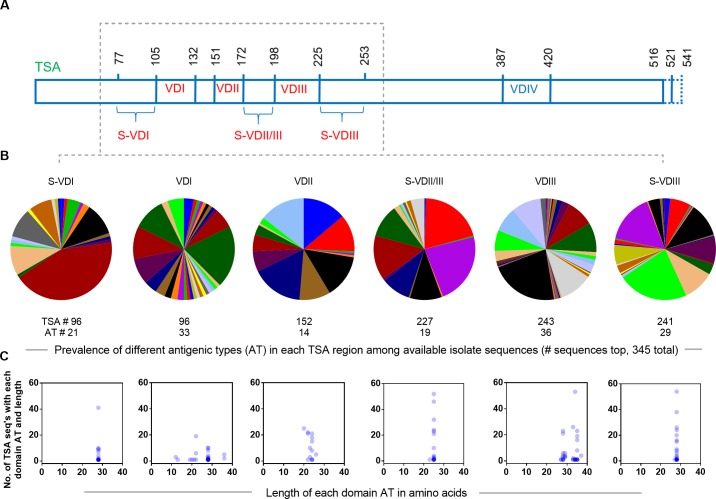
Structural illustration of TSA and prevalence and disparity of TSA ATs in *Orientia tsutsugamushi* from India. A) Schematic representation of TSA and the boundaries of different variable domains (VDI—VDIV) and spacer domains (S-VDI–S-VDIII) in the Gilliam prototype strain (NCBI accession # DQ485289, length 521 AA, which was used as a reference strain to align all the Indian TSA amino acid sequences. The lengths of available Gilliam type strains vary from 516–541 AA. B) 345 TSA sequences were studied; number of TSA with sequences covering each of the six domains ranged from 96–243. The pie diagrams show that the prevalence (percentage of TSAs with a given AT type) of different antigenic (sequence) types (AT) ranged from 14–36 in each domain region of Indian TSA sequences. The thinnest slices are from single sequences while thicker colors are from TSA types with the same sequence). C) The scatter plots show the length of each AT type in amino acids (X axis), and number of Ots strains present with a particular AT type (Y axis). The dark blue circle indicates that several ATs were present with the same length and numbers of sequences. Greater disparity in the AT lengths was observed among the three variable domains than the three conserved spacer domains.

The 345 TSA sequences Ots from India we analyzed in this study were retrieved from the NCBI GenBank (S1A Table). A similar set of 391 TSA sequences was obtained for Ots from South Korea as an outgroup comparison (S1B Table). Both of these TSA sets were aligned using MUSCLE [26]. The variable domains I, II, and III and the spacer domains (sequences between the variable domains) II and III (S-VDII/IIII) for all TSA sequences were identified as previously described [2, 13, 24] (Fig 1A). Similar size spacer regions before the VDI (S-VDI, 28 AA) and after VDII/III (S-VDIII, 29 AA) were independently extracted from the alignments to identify the extent and nature of mutations and antigenic variants found in adjacent less variable TSA regions. Antigenic types (AT) were initially defined very liberally as any unique TSA amino acid sequences found in the respective data sets regardless of the type of amino acid substitutions involved.

Independently extracted amino acid sequences for these regions were used to predict the CD4 TCEs with the Immune Epitope Database and Analysis Resource (IEDB) analysis resource consensus tool using IEDB recommended prediction method defaults [27, 28]. The spacer between VDI and VDII was too small for similar analysis of CD4 epitopes and VDIV was under represented among the partial Indian TSA sequences available.

### Prediction of binding affinity of T-cell epitopes to predominant Indian and South Korean MHC Class-II alleles

**Fig 2:**
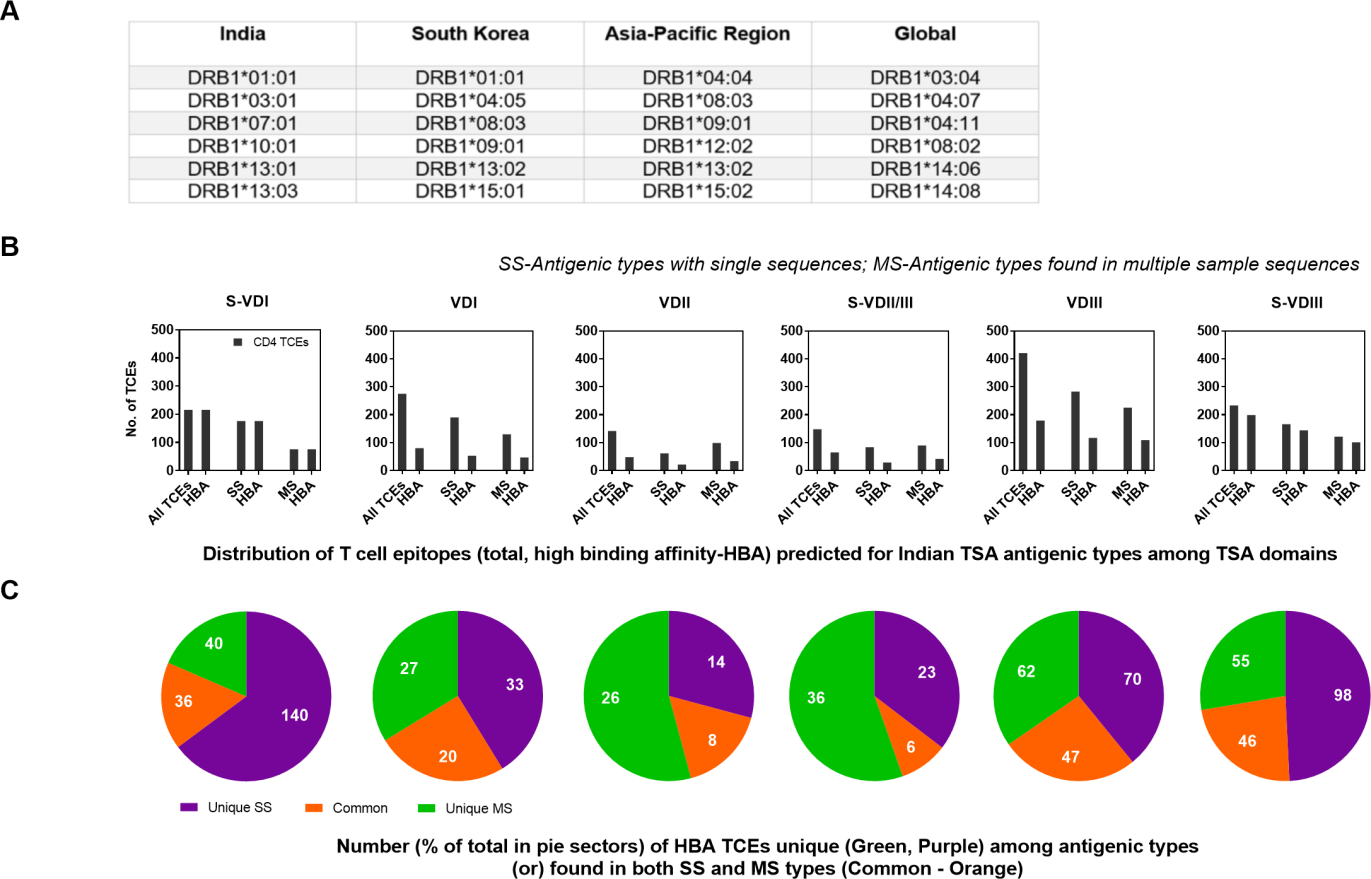
Prevalent HLA-DRB1 alleles in different geographical regions and domain prevalence of Indian 15-amino acid TSA peptides with predicted high binding affinities. A) Top six most prevalent HLA-DRB1 alleles identified in populations in India, South Korea, Asia-Pacific region and global (http://allelefrequencies.net/), which were used to predict the binding affinity of all predicted 15 aa peptides from each AT from six domains of Indian TSA. B) The distribution of HBA T-cell epitopes predicted from Indian TSA antigenic types in each TSA domain. For each domain, the first set of bars shows the total number of predicted unique peptides and the number of these peptides identified with HBA to at-least one prevalent Indian HLA allele. Both the total number of predicted peptides and the peptides with HBA were further classified based on their presence in AT detected in single (SS) or multiple strains (MS) (bar pairs 2 and 3, respectively). C) Distribution (percentages) of peptides (15 aa) with HBAs from ATs identified in single (SS-uniquely present) and multiple (MS-shared sequences ≥2) Ots strains.The peptides present in both single and multiple strain sequences are highlighted in orange, the HBA peptides in SS and MS types are highlighted in purple and green, respectively.

**Fig 3:**
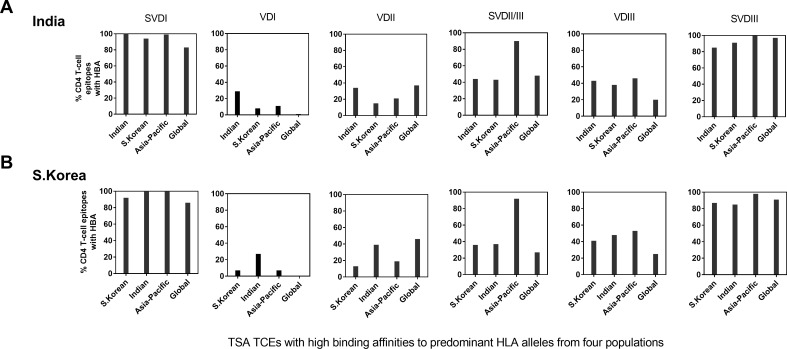
Proportion of peptides with HBA (CD4 TCEs) from the six domains of TSA from India and South Korea. The bar diagrams show that the proportion of predicted HBA peptides from all ATs from each of six domains from A) India TSA and B) South Korea TSA. Six prevalent HLA-DRB1 alleles from four different population sets (see Fig 2A) were used to identify the CD4 TSA TCEs with HBA.

For all potential CD4 TCEs from the Indian and Korean Ots, the binding affinity was predicted using the six most prevalent Human Leukocyte Antigen (HLA; MHC Class-II) alleles (http://allelefrequencies.net/) detected in India and South Korea, to identify the most immunodominant CD4 epitopes (15-mer) (Fig 2A). The HLA-DR alleles employed in this study covered most of the Indian population. The binding prediction results are given in units of IC 50nM, such that a lower number indicates higher affinity. Therefore, we considered only those peptides that were predicted to be bound by the given alleles with high binding affinity (HBA) score IC< = 10nM, as they are more likely to bind and stimulate an effective immune response. Since, the prevalence and nature of Ots TSA types and dominant HLA types may vary in human populations from different geographical areas, the prevalent HLA-DR alleles of three other populations (Korea, Asia-Pacific region) and globally (Fig 2A) have also been used for binding affinity predictions to assess the generality of those predicted HBAs (Fig 3).

**Fig 4:**
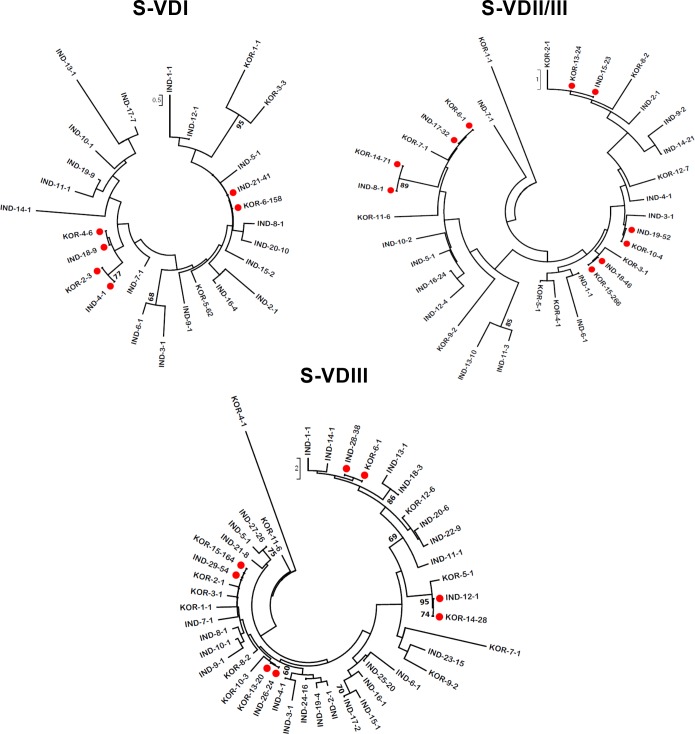
Phylogenetic relationships of Ots TSA spacer domains. All the ATs from each of three spacer domains S-VDI, S-VDII/III and S-VDIII of Indian and South Korean TSA were used for constructing individual Neighbor-Joining phylogenetic trees using their amino acid sequences. The TSA country of origin, the AT type and number of samples with that AT domain type are indicated for each unique AT type for each data set (S3 Table) (e.g., India-24-18). The pairs of identical 12 AT present in both India and South Korea TSAs are highlighted with red circles. Only the nodes with ≥70 bootstrap values are shown (1000 bootstraps). The bracket values show the scale for amino acid differences for each domain.

The Neigbor-Joining (NJ) phylogenetic trees were constructed in MEGA v7 [29] from the sequence alignments generated from the three spacer domain sets from both countries using MUSCLE program, to infer their phylogenetic relationships (Fig 4).

**Fig 5:**
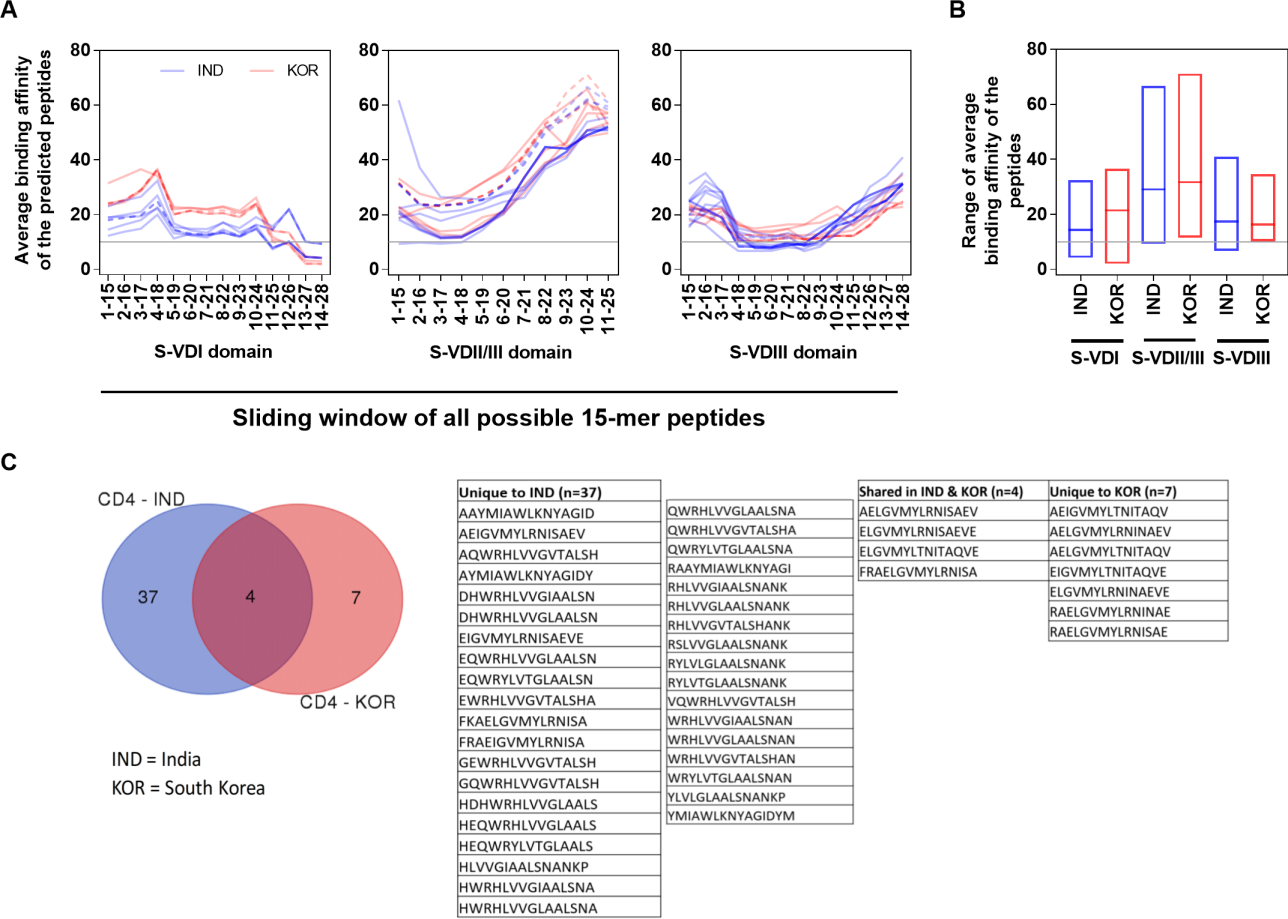
Properties of peptides exhibiting HBA from Indian and South Korean TSAs. A) The mean binding affinity score was calculated from the binding affinity (score) of each predicted 15 aa peptides (sliding window approach) from ATs present in multiple strains with six prevalent HLA types studied from each country. A total of 7 and 5 ATs in S-VDI, 11 and 8 ATs in S-VDII/III and 13 and 8 ATs in S-VDIII were present in multiple Ots TSA sample sequences from India (blue) and South Korea (red), respectively. The overlapping 7 identical ATs present in multiple Ots strains from both India (blue dash lines) and Korea (red dash lines) TSA spacer domains are also shown. B) The range and the median average binding affinities of the peptides predicted from three spacer domains. C) Comparison of unique HBA TCEs from Indian and South Korean TSAs.

We further characterized prevalent ATs present in multiple Indian and South Korean Ots strains to identify those most disparate immunochemically (using the BLOSUM62 matrix to exclude less important conserved amino acid changes) to further define the most immunologically significant epitopic regions (Fig 5) in the TSA spacer domains. We calculated the average binding affinity score of each predicted 15-mer peptide against all the predominant HLA types from India and South Korea using a sliding window approach. Peptides from each of 12 ATs in S-VDI (7 IND, 5 KOR), 21 ATs in S-VDIII (13 IND, 8 KOR) domains and 11 peptides from each of the 19 (11 IND, 8 KOR) ATs in S-VDII/III domain, were identified and used for calculating the average binding affinity against predominant HLA types from both India and South Korea. If the amino acid changes in the given epitope from a majority of antigenic types show higher binding affinity (IC< = 10nM) to the given HLA Class-II alleles than other changes, they are considered to be highly important amino acid sites, while others are less important amino acid sites. The range and median of these values were also calculated (Fig 5B). GraphPad Prism v7 was used for all simple statistical analyses and for drawing graphs to present our data.

## Results and discussion

### Impact of antigenic variations in the CD4 T-cell epitopes (TCE) on their high binding affinity (HBA) in both variable domains and spacer regions

The antigenic diversity of Ots has been a serious issue for developing effective diagnostic assays and a broadly effective vaccine for scrub typhus. Thus, we carefully analyzed the diversity of each of the six domains S-VDI, VDI, VDII, S-VDII/III, VDIII and S-VDIII (Fig 1). VDIV could not be analyzed since mostly only partial fragments of TSA from 345 Indian samples are reported in the database; these were derived almost exclusively by sequencing nested PCR amplicons obtained from scrub typhus patient samples. These six TSA domains (Fig 1A) were analyzed to predict the immunodominant CD4 epitopes that might be suitable targets for sub-unit vaccine development for Indian populations. By amino acid alignment, a total of 33, 14, and 36 potentially different unique antigenic types (ATs) were present in highly dispersed variable domain I (VDI; 12–36 AA), VDII (20–26 AA), VDIII (27–37 AA), while conserved spacer domainsS-VDI, S-VDII/III and S-VDIII contained 21 (28 AA), 19 (23–25 AA) and 29 (27–28 AA) ATs, respectively (Fig 1B and 1C). Among 152 ATs, 2 in VDI had a lengths of 12–13 AA and therefore are restricted to CD8 epitopes, while the remaining 150 ATs with ≥15 AA (range 19–37 AA) in length are typical of potential CD4 TCEs (Fig 1C). The total number of ATs (n = 150) identified from Indian TSA is 2-fold higher than the ATs (n = 75) reported for South Korean TSA (S1 Fig), indicating that the available TSA domains from South Korea are more conserved. While both CD4 and CD8 responses are important for the elimination of intracellular bacteria like *Orientia* [30–36], we focused our study on this more robust CD4 data.

All the 150 ATs with ≥15 AA were used to obtain the 15-mer peptide datasets and calculate their binding affinities to prevalent Indian HLA types (Fig 2A). The three spacer variable domains S-VDI, S-VDII/III and S-VDIII had a comparatively high proportion (44–100%) of peptides (CD4 TCEs) which had the highest predicted binding affinity/energy (HBA, IC50 value ≤10 nM) to at least one of the Indian HLA types analyzed, while TCEs from variable domains VDI-III (29–43%) had fewer (Fig 2B). Notably, 100% of the predicted peptides in the S-VDI had HBAs to at least one Indian HLA type. Overall, 55% (787/1425) of the total possible TCEs represented had HBAs against the predominant Indian HLA-DR alleles; these are immunodominant CD4 TCEs, which can be predicted to mount effective immune responses in populations in India. The spacer S-VDI, S-VDII/III and S-VDIII regions, had a higher proportion (61%, 480/787) of predicted CD4 TCEs with HBAs than those found in the variable domains VDI-III (39%, 307/787). This suggests that the conserved spacer domains S-VDI, S-VDII/III and S-VDIII may be more important in immune responses to *Orientia* infections than previously appreciated. Among the 787 TCEs with HBAs, 48% and 31% of TCEs occurred in antigenic types found in single sequences (378; purple) or multiple times (246; green), respectively, while the remaining 21% (163) TCEs are present in antigenic types found in both unique and shared sequence types (orange) (Fig 2C); the 52% of HBA TCEs present widely in myriad isolates are probably most important for inducing immune responses in the majority of the population in India, assuming that these isolates are indeed sufficiently representative of the range of Ots TSA types found in India.

### Binding affinity of TSA CD4 TCEs to HLA-DR alleles prevalent outside of India: South Korea, Asia-Pacific region and across different continents

In order to evaluate whether similar CD4 HBA T cell epitopes are present on the different antigenic types of TSA found in different countries and recognized by different HLA types (Fig 2A), we first compared the Ots TSA HBA domains identified from the Indian HLA types to the Korean TSA HBA sites predicted for dominant HLA types from South Koreans and vice versa (Fig 3). It should be noted that South Korea has a much smaller geographic area than India and has a high predominance of Boryong type throughout the country. Similarly, we also evaluated the binding of prevalent HLA-DR types from other human populations in the Asia-Pacific region and more globally (all continents) to TSA sequences available for both the Indian and Korean types of Ots, as some of the Indian dominant antigenic types are also found in other countries from the Asia-Pacific region (Fig 3). Overall, ≥83% of predicted peptides from spacer domains S-VDI and S-VDIII were predicted to have HBA against prevalent HLA types from Indian, South Korean, Asia-Pacific region and global population data sets, while predicted peptides with HBA from S-VDII/III domain showed 27–92% of CD4 TCEs with HBA; the maximum proportion of 92% was observed against Asia-Pacific HLA types (Fig 3A and 3B, S2 Table). In contrast only a total of ≤53% of peptides in the three variable domains (VDI-III) exhibited HBA.

A considerable proportion of TCEs (43–58%) from all six domains from Indian TSA had HBA to HLA alleles that are less prevalent or not reported from India, but more prevalent in the other three geographical regions; this finding suggests that these HBAs are important for inducing optimal immune responses worldwide. Similar results were observed for CD4 HBA TCEs from all domains of South Korean TSA (45–59%) with HLA types found in populations other than South Koreans (Fig 3, S2 Fig). These results indicate that even though different Ots ATs and HLAs are found in India and South Korea, the binding pattern of CD4 TCEs in all six TSA domains are similar. Interestingly, a higher proportion (58% with Indian TSAs and 60% with South Korean TSAs) of TCEs with HBA, respectively were identified with the Asia-Pacific population HLA types with all TSA domains. In summary, our results indicate that a larger proportion of CD4 HBA TCEs from spacer domains than those from variable domains appear important for mounting strong immune responses in the four sets of populations that we studied with either Indian or Korean TSA TCEs.

### Evolutionarily and functionally important amino acid changes in the regions of the TSA spacer domains

Since our analyses showed that the conserved spacer domains S-VDI, S-VDII/III and S-VDIII may be more important in immune responses to Ots infections than previously appreciated, we further analyzed them to infer i) the phylogenetic relationships of the TSA found in Ots circulating in both India and South Korea by TSA domain, and ii) the evolutionarily and immunologically most significant peptide regions within the three spacer domains. Although more AT domain clades with unique sequences are found in the more diverse TSA data from India than Korea (cf. Fig 1, S1 Fig), the distribution of related isolates is generally not isolated geographically or by prevalence for any of these three spacer domains (Fig 4). Indeed, 12 identical ATs (highlighted in red circles) including 3 in S-VDI, 5 in S-VDII/III and 4 in S-VDIII were present in ATs from both Indian and South Korean patients. The key amino acid changes R21T and S24T in S-VDI were significant in separating ATs into two major clades (S3 Fig) and many of the other closely domain types were distinguished by conserved aliphatic amino acid differences. Similarly, amino acid changes C12Y and R18M in S-VDII/III and H2L amd T15A in S-VDIII drove the separation of ATs into two broad clades (S3 Fig, S3 Table). These current dichotomies may prove somewhat simplistic for S-VDI and S-VDII/III as more complete TSA sequences from more sites are obtained in India and in other countries but also appear significant for TSA domains from other countries.

In a second approach, we further characterized the immunochemistry of the most prevalent spacer domains of the ATs present in multiple Indian and South Korean Ots strains. We calculated the average binding affinity score of each of the predicted 15-mer peptides against each of the predominant HLA types from India and South Korea using a sliding window approach. The overall binding affinity scores ranged from 2.0–36.6 (median 15.7), 9.3–71.1 (30.9) and 6.7–41.0 (16.3) for the three domains S-VDI to S-VDIII, respectively from both countrires (Fig 5A). The three spacer domains from Indian and South Korean TSA were also analysed individually, which showed that the median average binding affinity score of peptides from S-VDI and S-VDII/III of Korean TSA is significantly (p-value <0.0001, Kruskal-Wallis test) higher (lower binding) than the score observed from Indian TSA for these domains (Fig 5B). Peptides from domains S-VDI and S-VDIII had a greater number of peptides with high binding affinity scores as confirmed by the mean range and distribution plots than the S-VDII/III domain (Fig 5B).

Region 11–28 (18 AA) of 7 and 5 ATs in S-VDI from India and South Korea, respectively, had 15 (8 unique) and 14 (11 unique) CD4 HBA TCEs, respectively (Fig 5A, 5B and 5C, S4 Table). Interestingly, only region 1–18 (18 AA, 4 TCEs) from a single Indian AT in S-VDII/III and region 4–23 (20 AA, 49 [29 unique] CD4 TCEs) of 12 ATs in S-VDIII from Indian TSA were identified to be functionally important regions with HBA to all the HLA types studied, but none were observed from Korean TSA in S-VDII/III or S-VDIII. In S-VDI domain, ATs IND-15-2, IND-16-4, IND-20-10 and IND-21-41 had the hydrophilic amino acid change T21R that was important in predicted strong HBA for the TCEs carrying arginine. Similar results were observed in Korean ATs KOR-3-3, KOR-5-62 and KOR-6-158. In S-VDII/III, AT IND-10-2 showed hydrophilic/neutral amino acid changes R/E/E/A4Y leading to improved HBA for the TCEs carrying tyrosine. In S-VDIII, AT IND-23-15 had hydrophobic amino acid changes L17M and AT IND-29-54 had H9S (neutral/hydrophilic) which had minimal predicted functional impact, as the TCEs carrying methionine and serine exhibited low binding affinity to Indian alleles (S3 Fig). A third small branch of S-VDIII was chemically related to clade II (Fig 4, S3 Fig) and grouped with it in identifying the important HBA CD4 TCEs (S4 Table). Notably, 7 identical ATs present in multiple Ots strains from both India (blue dash lines) and Korea (red) TSA spacer domains (Fig 5A) helped confirm or refute the significance of differences in the pattern of binding affinity, since the peptides from these ATs were mapped against two different sets of prevalent alleles from two different countries and confirmed that identical peptides do exhibit different binding interaction to different HLA domains (Fig 5A and 5B). It also clearly indicates that only a small number of the spacer domain peptides are predicted to be uniformally important in different ATs in inducing strong host immune responses. While peptide regions in a few conserved domains may stimulate the host immune responses actively, much of the other variable regions of the TSA protein appear to favor the ability of Ots to evade host protective immunity.

Mutations in the TSAs that we identified can potentially strongly impact binding to specific HLA-DRB1 alleles. The point mutations, indels and genetic exchange occurring among different TSA genotypes probably also contribute significantly to Ots genetic diversification [17, 24]. However, this is probably driven by selection in vector and host mammalian immune systems rather than in humans because scrub typhus is a dead end infection in people. One limitation of this study is the extensive usage of partial fragment sequences of TSA from clinical samples that are used for genotyping, so acquisition of complete or nearly complete TSA gene sequences might be required to more clearly define Ots genetic and antigenic types completely, especially for VDIV and adjacent more conserved sites in TSA. Our analysis suggests that the conserved S-VDI, S-VDII/III and S-VDIII spacer domains, especially the functionally important regions from S-VDI and S-VDIII are probably more important in immune responses to Ots Infections than previously appreciated. However, even those domains have been subjected to diversification, thus making vaccines focused on those sites difficult to produce. Our analysis suggests that such a novel scrub typhus vaccine would need to consider including a number of these 41 immunodominant TSA CD4 epitopes (Fig 5C, S4 Table) to induce an efficacious T-cell response in Indian populations. Because the number of shared CD4 HBA TCE between Indian and Korean TSA is very small, a broadly reactive vaccine seems very unlikely. This finding is consistent with the substantial problems experienced to date in developing a good TSA vaccine with heterologous efficacy in mouse models of infection. Differences in TSA at the epitope level may contribute to the wide range of pathogenicity of Ots strains in humans and rodent and chigger infection challenge models. Direct experiments are needed to confirm further the immunological significance and contribution of these variant domains in TSA T-cell functional assays and their role in clearance of Ots from infected hosts.

## Supporting information

S1 Fig The prevalence and diversity of TSA ATs in *Orientia tsutsugamushi* from South Korea. A) The pie diagrams (percentage of TSAs with a given AT type) and data numbers show that from a total of 391 TSA sequences studied the number of sequences from each of the six domains studied ranged from 233–390 and these all contained only 6–19 ATs, thus demonstrating the greater homogeneity of South Korean isolate TSA data than that from India TSA. B) The scatter plots show the length of each AT type in amino acids (X axis), and number of Ots strains present with a particular AT type (Y axis). The dark blue circle indicates that several ATs were present with same length and numbers of sequences. Greater disparity in the AT lengths was observed among the three variable domains than the three conserved spacer domains.
(TIF)

S2 Fig The domain prevalence of South Korean 15 amino acid TSA peptides with predicted high binding affinities. A) In the bar diagram for each domain, three sets of bars are shown; the first set shows the total number of peptides predicted and the number of predicted HBA peptides (CD4 TCEs). The other paired bars show the total number of predicted peptides and HBA peptides further classified based on their presence in AT identified as present in single or multiple Ots TSA sequence samples. B) The pie diagrams show that distribution of the HBA peptides from ATs identified in single (SS) and multiple Ots strains (MS) from Korea. While the peptides present in both single and multiple strains were highlighted in orange, the peptides that were unique to SS and MS types highlighted in purple and green, respectively.
(TIF)

S3 Fig Multiple sequence alignments of ATs from three spacer domains of TSAs from both India and South Korea. S-VDI: a total of 27 ATs detected, in which 12 ATs are present in multiple strains; S-VDII/III: a total of 34 ATs detected, in which 19 ATs are present in multiple strains; S-VDIII: a total of 44 ATs detected, in which 21 ATs are present in multiple strains. The AT clade subgroupings for each domain are indicated. The key amino acid changes are indicated with red arrows.
(TIF)

S1 Table Strain name and the accession number of TSA sequences of Ots from A) India (n = 345) and B) South Korea (n = 391) analysed in this study.
(XLSX)

S2 Table Details of the number of predicted peptides with HBA (CD4 TCEs) from each of six domains of TSA from A) India and B) South Korea, against four different sets of predominant HLA alleles (please refer to Fig 2A).
(XLSX)

S3 Table Ots strains studied from A) India and B) South Korea, and their designated spacer domain antigenic type.
(XLSX)

S4 Table Peptide sequences of high confidence HBA CD4 T-cell epitopes from functionally important regions of the three spacer domains of TSA from India and South Korea TSAs. (XLSX)
