# Supplementary figures and images for: Complexity of type-specific 56 kDa antigen CD4 T-cell epitopes of Orientia tsutsugamushi strains causing scrub typhus in India
Source: PLoS One. 2018 Apr 26;13(4):e0196240. doi: 10.1371/journal.pone.0196240 (PMC5919512; doi:10.1371/journal.pone.0196240)

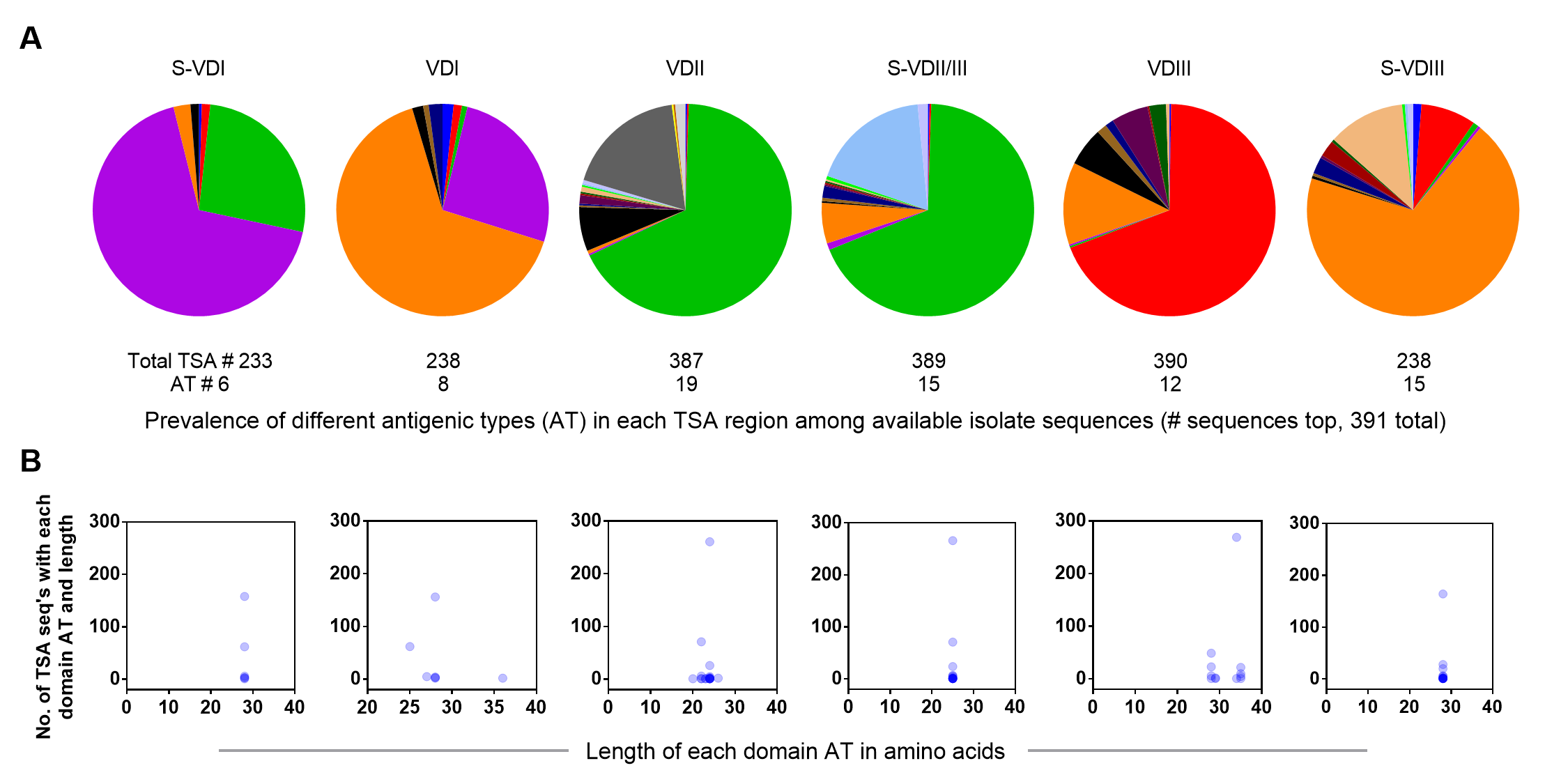

Supplement: S1 Fig — A) The pie diagrams (percentage of TSAs with a given AT type) and data numbers show that from a total of 391 TSA sequences studied the number of sequences from each of the six domains studied ranged from 233–390 and these all contained only 6–19 ATs, thus demonstrating the greater homogeneity of South Korean isolate TSA data than that from India TSA. B) The scatter plots show the length of each AT type in amino acids (X axis), and number of Ots strains present with a particular AT type (Y axis). The dark blue circle indicates that several ATs were present with same length and numbers of sequences. Greater disparity in the AT lengths was observed among the three variable domains than the three conserved spacer domains. (TIF) [file pone.0196240.s001.tif]

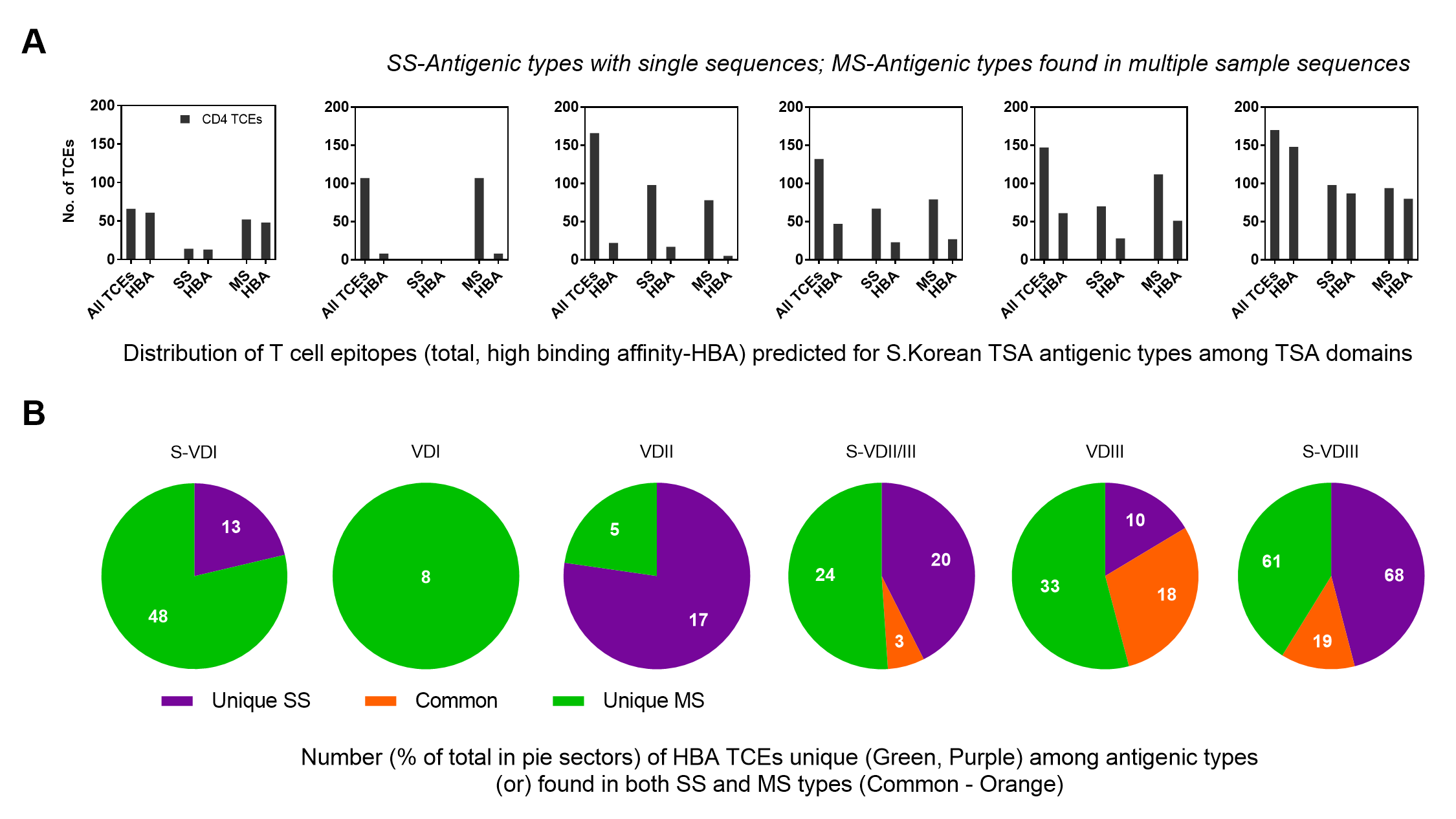

Supplement: S2 Fig — A) In the bar diagram for each domain, three sets of bars are shown; the first set shows the total number of peptides predicted and the number of predicted HBA peptides (CD4 TCEs). The other paired bars show the total number of predicted peptides and HBA peptides further classified based on their presence in AT identified as present in single or multiple Ots TSA sequence samples. B) The pie diagrams show that distribution of the HBA peptides from ATs identified in single (SS) and multiple Ots strains (MS) from Korea. While the peptides present in both single and multiple strains were highlighted in orange, the peptides that were unique to SS and MS types highlighted in purple and green, respectively. (TIF) [file pone.0196240.s002.tif]

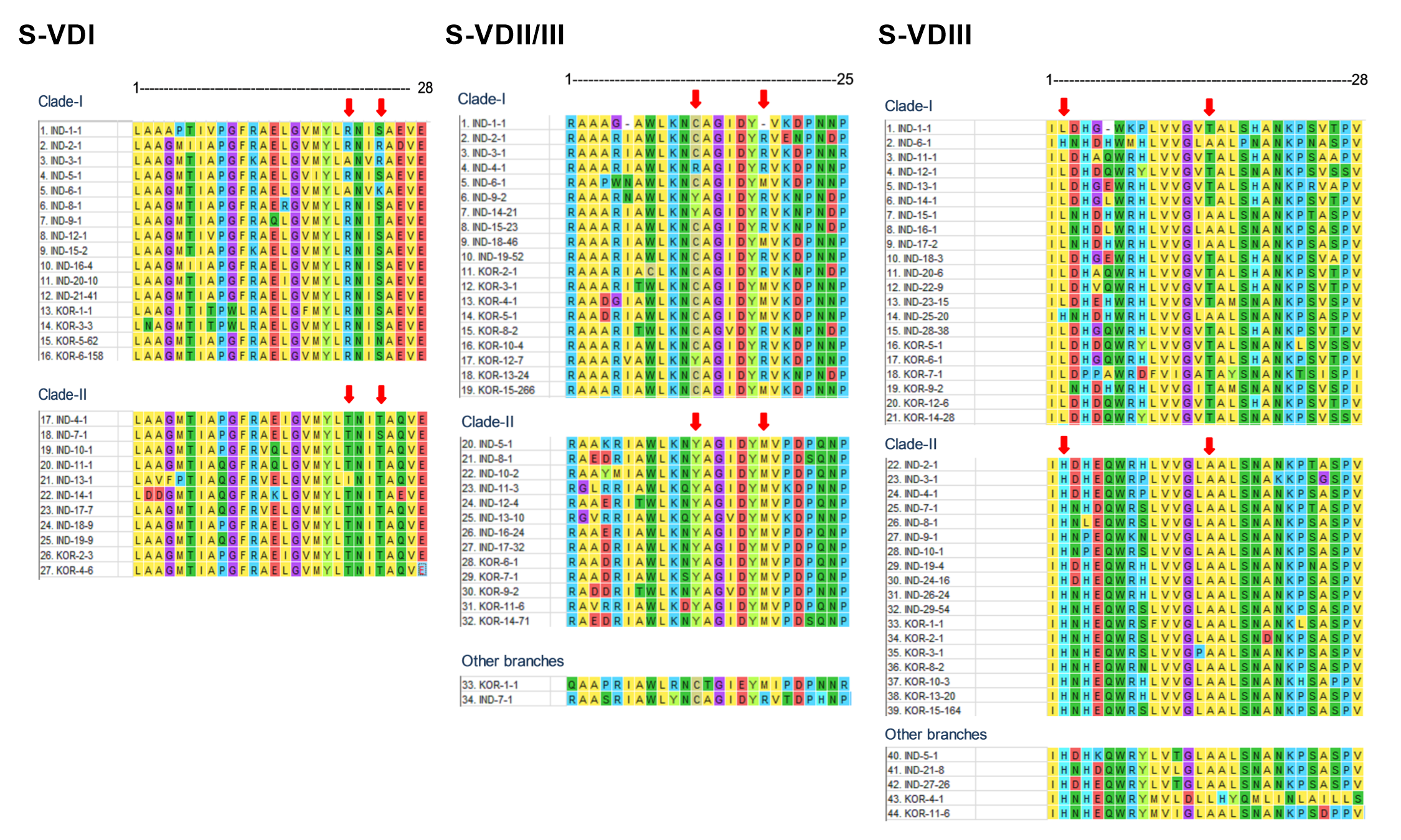

Supplement: S3 Fig — S-VDI: a total of 27 ATs detected, in which 12 ATs are present in multiple strains; S-VDII/III: a total of 34 ATs detected, in which 19 ATs are present in multiple strains; S-VDIII: a total of 44 ATs detected, in which 21 ATs are present in multiple strains. The AT clade subgroupings for each domain are indicated. The key amino acid changes are indicated with red arrows. (TIF) [file pone.0196240.s003.tif]
